# Modeling of Microsegregation and Homogenization of 6xxx Al-Alloys Including Precipitation and Strengthening During Homogenization Cooling

**DOI:** 10.3390/ma12091421

**Published:** 2019-05-01

**Authors:** Panagiota I. Sarafoglou, Alexandros Serafeim, Ioannis A. Fanikos, John S. Aristeidakis, Gregory N. Haidemenopoulos

**Affiliations:** 1 Department of Mechanical Engineering, University of Thessaly, 38334 Volos, Greece; pa.sarafoglou@gmail.com (P.I.S.); johnfanikos@gmail.com (I.A.F.); iaristeidakis@gmail.com (J.S.A.); 2 RWTH Aachen, Institut für Eisenhüttenkunde, Intzestrasse 1, 52072 Aachen, Germany; Alexandros.Serafeim@iehk.rwth-aachen.de; 3 Department of Mechanical Engineering, Khalifa University, Abu Dhabi 127788, UAE

**Keywords:** microsegregation, homogenization, aluminum alloys, precipitation, strengthening, homogenization cooling

## Abstract

Control of the homogenization process is important in obtaining high extrudability and desirable properties in 6xxx aluminum alloys. Three consecutive steps of the process chain were modeled. Microsegregation arising from solidification was described with the Scheil–Gulliver model. Dissolution of Mg_2_Si, Si (diamond) and β-AlFeSi (β-Al_5_FeSi) to α-AlFeSi (α-Al_12_(FeMn)_3_Si) transformation during homogenization have been described with a CALPHAD-based multicomponent diffusion Dual-Grain Model (DGM), accounting for grain size inhomogeneity. Mg_2_Si precipitation and associated strengthening during homogenization cooling were modeled with the Kampmann–Wagner Numerical (KWN) precipitation framework. The DGM model indicated that the fractions of β-AlFeSi and α-AlFeSi exhibit an exact spatial and temporal correspondence during transformation. The predictions are in good agreement with experimental data. The KWN model indicated the development of a bimodal particle size distribution during homogenization cooling, arising from corresponding nucleation events. The associated strengthening, arising from solid solution and precipitation strengthening, was in good agreement with experimental results. The proposed modeling approach is a valuable tool for the prediction of microstructure evolution during the homogenization of 6xxx aluminum alloys, including the often-neglected part of homogenization cooling.

## 1. Introduction

The process chain of extrudable 6xxx aluminum alloys consists of a series of individual elements including melting and alloying, direct-chill casting in billets, homogenization, extrusion and finally, aging. From the aforementioned list, homogenization and the associated phase transformations, have attracted increased attention in the recent years due to the importance of the homogenization process in obtaining high extrudability and desirable properties in the extruded profiles. The as-cast material contains microstructural inhomogeneities, arising from the solidification process, which include elemental microsegregation, in the level of the secondary dendrite arms, grain boundary segregation, and the formation of several low-melting eutectics and intermetallic compounds, which degrade the extrudability of the as-cast billet [1,2].

Among the intermetallics, the Fe-bearing intermetallics are the most important, α-Al_12_(FeMn)_3_Si and β-Al_5_FeSi, from now on called α-AlFeSi and β-AlFeSi, respectively. The α-AlFeSi has a cubic crystal structure and globular morphology while the β-AlFeSi exhibits a monoclinic structure and a plate-like morphology, which limits the extrudability of the as-cast billet by inducing local cracking and surface defects in the extruded material [1,3,4]. These effects can be partially or completely eliminated with the application of a suitable homogenization treatment. The benefits of this treatment include the following: removal of elemental microsegregation, dissolution of low-melting eutectics, transformation of iron-containing intermetallic compounds, shape control (round-off) of hard particles with sharp edges, dissolution of the grain-boundary Mg_2_Si phase, and re-precipitation with a more homogeneous in-grain distribution during homogenization cooling. All these effects improve the extrudability and increase the response of the material to natural or artificial aging [5].

The most important literature data concerning experimental and modeling studies are presented below.

Regarding experimental studies, the homogenization process has been by several researchers. Specific topics of interest are the dissolution of Mg_2_Si, the β-AlFeSi→α-AlFeSi transformation and the re-precipitation of Mg_2_Si during homogenization cooling. These studies are discussed below. The homogenization process has been studied experimentally by several researchers. The microstructure evolution of the 6063 alloy during homogenization has been studied, for various thermal cycles, in [6]. The dissolution of Mg_2_Si has been followed with electrical resistivity measurements, while the distribution of the alloying elements has been investigated with electron microprobe analysis for the 6061, 6069 alloys [7]. The dissolution and coarsening kinetics of the Mg_2_Si precipitates during reheating of the homogenized material, prior to extrusion, has been discussed in [8,9]. Microstructural changes during homogenization have been discussed for a 6063 alloy in [10], including the β-AlFeSi→α-AlFeSi transformation. The α-AlFeSi phase undergoes morphological changes, which involve rounding of edges, pinching, and necklace formation. These changes have been described in [11,12,13] while a quantitative description of the progress of the homogenization, based on microstructural indices, such as the aspect ratio and circularity, was recently presented in [14]. Secondary precipitation has been followed with high-temperature flow stress measurements in [15]. During the homogenization cooling, the Mg_2_Si phase re-precipitates and forms a new dispersion. The particle size distribution (PSD) of this dispersion is an important parameter influencing extrudability. Several authors studied the influence of the cooling rate after homogenization on the alloy microstructure. The influence of the cooling rate on the final mechanical properties for the 6063, 6082, and 6005 alloys has been investigated in [16,17] while the influence of the cooling rate on the extrusion speed for various chemical compositions of Al-Mg-Si alloys is discussed in reference [18]. Precipitation during homogenization cooling, including the effect of cooling rate, has also been discussed in [19,20,21]. It appears that the precipitation of the metastable β′-Mg_2_Si phase instead of the equilibrium β-Mg_2_Si phase enhances the extrudability of the material [16].

Regarding simulation studies and due to the importance of designing a suitable homogenization process, several modeling approaches, regarding specific aspects of homogenization, have been developed and reviewed recently [22]. The development of microsegregation, including the mapping of phase fractions of Mg_2_Si and β-AlFeSi versus alloy composition, has been recently discussed in [23,24]. Modeling of the homogenization process is discussed in detail in [25,26,27]. The transformation of β-AlFeSi→α-AlFeSi has been modeled for short homogenization times in [28], while the effect of Mn and Si on the kinetics of the transformation is discussed in [29,30]. The complex interaction of Fe and Mn has been investigated with a diffusion model in [31]. Microstructure evolution during homogenization cooling has been modeled in [32] while a numerical model treating both homogenization holding and homogenization cooling has been presented in [33]. The aforementioned modeling approaches treat individual elements of the process chain. In contrast, the proposed modeling approach treats three consecutive elements of the process chain i.e. microsegregation during solidification, homogenization and homogenization cooling, in an effort to provide insight into the effect of as-cast grain size inhomogeneity as well as precipitation and associated strengthening during homogenization cooling.

## 2. Materials and Experimental Procedures

The alloys considered in this study are shown in Table 1, where the chemical compositions listed were obtained by optical emission spectroscopy. The work on the modeling of microsegregation and homogenization was performed on alloy 6082, while the work on homogenization cooling was performed on all three alloys. Billets from the three alloys with diameter 200 mm were prepared with direct-chill casting. For the 6082 alloy, one billet was used for the study of microsegregation of the as-cast condition. Homogenization was performed at 560 °C for 0.5, 4, 8, and 32 h. For the study of homogenization cooling, specimens from the as cast billets of 6063, 6005, and 6082 alloys with dimensions 14 cm × 2 cm × 2 cm were cut and homogenized at 580 °C for 8 h. The specimens were cooled in air and the temperature was monitored with a thermocouple attached to the specimens.

For metallographic investigation, the billets were sliced and specimens were cut in the transverse direction to the billet axis. Specimen preparation included cutting and grinding with SiC papers rating 120, 320, 500, 800, 1000, and 2400 grit. Polishing was performed with 1 μm diamond paste followed by electro-polishing with Barkers solution consisting of 10 mL fluoroboric acid (35%) and 200 mL water. The microstructure was revealed after etching in Keller’s solution, consisting of 0.5% HF in 50 mL H_2_O in order to reveal the intermetallic phases. Metallographic examination was carried out using an optical microscope (Leitz Aristomet, Wetzlar, Germany). Quantitative determination of volume fractions was performed using image analysis methods, based on point counting, using a 1000 grit on 10 sections according to the ASTM E-562 standard [34]. The intermetallic phases were first identified in the scanning electron microscope (SEM) by determining their chemical composition by energy dispersive spectroscopy (EDS), (JEOL JSM-6510, Tokyo, Japan). Similar metallographic procedures were applied to the homogenized specimens. X-ray diffraction analysis was carried out in order to monitor the evolution of intermetallic phases during homogenization. The analysis was performed with a Siemens D-5000 diffractometer (Siemens, Munich, Germany) operating with a Cu-Kα radiation. The sampling rate during the measurement was 0.008 °C/s for a total time of 4020 s.

Specimens for tensile testing were prepared from the samples of the three alloys subjected to homogenization cooling, according to ASTM E8M [35] specification. Tensile testing was performed on an INSTRON 8801 servo-hydraulic machine (Instron, Norwood, MA, USA) at a strain rate of 10^−4^ s^−1^.

## 3. Modeling Approaches

### 3.1. General Description

As mentioned in the introduction, the present work deals with the modeling of microsegregation during solidification, the homogenization holding and homogenization cooling. The processes investigated, the relevant phenomena and the models employed for the simulation are shown in Table 2.

Microsegregation of elements and phases was treated with the Scheil solidification model. The dissolution of Mg_2_Si and the transformation of β-AlFeSi to α-Al(FeMn)Si are treated with a Dual Grain Model (DGM) based on multicomponent diffusion in dispersed-phase systems. Finally, the precipitation of Mg_2_Si during homogenization cooling was treated with the Kampmann-Wagner-Numerical model, while the associated strengthening was treated with a relevant strength model. All these modeling approaches are described in the next sections.

### 3.2. Microsegragation During Solidification

Solidification during direct-chill casting employed in 6xxx alloys was assumed to take place under the condition of limited diffusion in the solid and infinite diffusion in the liquid (Scheil–Gulliver condition). This is an acceptable assumption since the local solidification times encountered in industrial direct chill casting are short and the diffusion coefficients of the alloying elements in the liquid phase are significantly larger than those in the solid phase. All calculations were performed with the Thermo-Calc 2015a software [36], which is based on the CALPHAD approach [37]. The use of CALPHAD approach in lightweight metallic alloys, including Al-Mg-Si-Fe-Mn alloys has been recently described in [38]. The database employed was the TCAL5 Al-alloy database, which has been recently described in [39]. It should be mentioned that other computational methods, which take into account macroscopic transport phenomena (melt flow and transport of crystals) have been developed for ternary alloys [40,41]. However, these methods use locally linearized phase diagrams and do not benefit, at the moment, from the available thermodynamic databases.

### 3.3. Homogenization (Holding)

**Figure 1:**
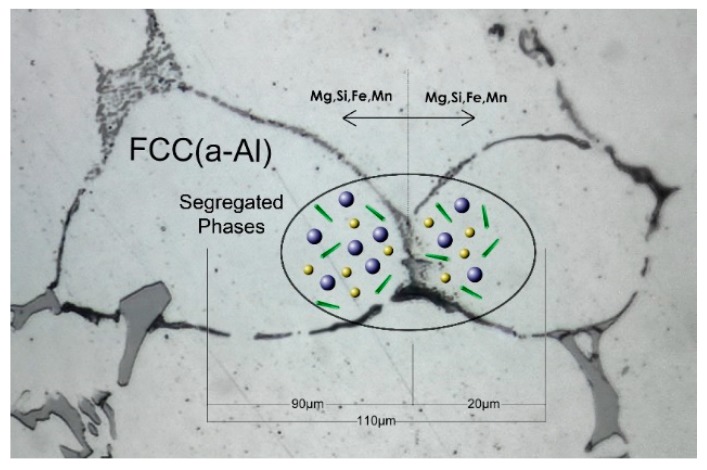
Calculation domain of the Dual-Grain Model consisting of two half-grains with sizes 90 and 20 μm. Total region size is 110 μm.

The model employed for the homogenization process is a modification of the model described in [30]. The main modification arises from the fact that the grain size in the as-cast microstructure is not uniform. The grain size inhomogeneity was taken into account by developing a Dual-Grain Model (DGM), which incorporates a calculation domain comprising of two neighboring grains, in contact, with significantly different grain sizes. In this way the higher transformation rate in the smaller grains was taken into account. The DGM region then serves as the representative volume element (RVE) for the solution of the homogenization problem. The DGM calculation domain is depicted in Figure 1. Following metallographic examination, two sizes of 180 μm and 40 μm respectively were selected for the construction of the DGM. Due to the symmetry, only half of the grains were considered. The total region size is 110 μm considering the two half-grains in contact (90 and 20 μm, respectively).

The composition as well as the phase fractions profiles, which were introduced as initial conditions in the DGM, were the Scheil microsegregation profiles of the as-cast microstructure, calculated from the relevant Scheil simulations. To that end, the fraction solid $f_{s}$, derived from Scheil was converted to a distance axis via the relation

(1) $$x_{s}=f_{s}L$$

where L is the region size of each grain (90 or 20 μm) in the DGM. The initial distribution of intermetallic phases, required for diffusion simulations, was obtained by differentiating the phase profiles obtained by Scheil simulations, with respect to the diffusion distance. Thus the diffusion problem was solved in one dimension. Three key phase transformations were treated concurrently in both grains of the DGM. These are the Mg_2_Si and Si (diamond) dissolution as well as the β-AlFeSi to α-AlFeSi transformation. Both are diffusional transformations and their rate is controlled by the diffusion of alloying elements. In the DGM model, only lattice diffusion through the matrix has been considered. Grain boundary diffusion or dislocation assisted diffusion were not considered, as in the homogenization process, diffusion takes place primarily from the boundaries to the grain interiors in order to eliminate the microsegregation gradients. In addition, the dislocation density of the material has nominal values corresponding to the annealed material, since the material has not undergone any cold deformation before homogenization.

The Mg_2_Si phase as well as the intermetallics α-AlFeSi and β-AlFeSi were considered as dispersed phases in the FCC matrix. One basic model assumption is that the growth and dissolution rates of the precipitate phases are very high compared with the diffusion rates in the matrix. Alternatively, the matrix diffusion is the controlling mechanism of the overall kinetics. This assumption is tolerable for the high homogenization temperatures, since the growth–dissolution rates are very high and the particles reach the equilibrium state very fast. Consequently, the local conditions are rather concentration dependent than time-dependent. Locally, most of the solute is trapped into the particles, reducing the concentration gradients and the diffusion rate in the matrix. The diffusion problem was solved with the computational kinetics package Dictra [36]. Use was made of the Dispersed Phase Module in Dictra. The module treats problems involving diffusion through microstructures containing dispersed precipitates or secondary phases. Diffusion is assumed to take place only in the matrix phase. The dispersed phases are considered as “non-diffusion phases”. They act as point sinks or sources of solute atoms in the simulation and their fraction is calculated from the local composition in each node, assuming local equilibrium. The growth or dissolution of phases leads to adjustments in the concentration profiles of the elements to be used in the next time-step of the calculation. What changes during the calculations in each time step is the volume fraction of phases and the local matrix compositions (composition profiles) through the matrix diffusion. As stated above, the diffusion problem is one-dimensional and is treated in a multicomponent (Al-Mg-Si-Fe-Mn) and multiphase system. The phases present in the system are the FCC aluminum matrix and the dispersions of Mg_2_Si, the iron intermetallics β-AlFeSi and α-AlFeSi, as well as pure Si particles in the form of a Si (diamond) phase. The solution of the diffusion equation is performed under the following boundary and initial conditions. Considering a closed system, the boundary conditions are:

(2) $$J_{i}(0,t)=J_{i}(L,t)=0$$

where $J_{i}$ are the elemental fluxes with *i =* Mg, Si, Fe, Mn. In terms of concentration gradients, the above equation becomes

(3) $${\frac{\partial c_{i}}{\partial x}|}_{{}^{\begin{array}{c} x=0\\ x=L \end{array}}}=0$$

The initial conditions for solving the diffusion problem are the results of the Scheil calculations, converted over the diffusion distance using Equation (1), for *t* = 0 and can be expressed as follows for the elements:

(4) $$c_{i}(x,0)=c_{i}^{s}(x)$$

while for the phases

(5) $$f_{k}(x,0)=\frac{\partial f_{k}^{s}}{\partial f_{S}}\Leftrightarrow \frac{1}{L}\int_{0}^{x}f_{k}(s,0)ds=f_{k}^{S}(f_{S}(x))$$

where *k* = Mg_2_Si, α-AlFeSi, β-AlFeSi, Si (diamond) and $c_{i}^{s}(x)$ & $f_{k}^{s}(x)$ are the composition profiles and phase fractions respectively, resulting from the Scheil simulation. It should be noted that in DICTRA the diffusivities are products of mobilities and corresponding thermodynamic factors. The mobility parameter, $M_{i}$ for an element *i* in a given phase is described by a frequency factor $M_{i}^{0}$ and activation energy $\Delta G_{i}^{*}$, which are related by the following equation:

(6) $$M_{i}=\frac{M_{i}^{0}}{RT}\exp(-\frac{\Delta G_{i}^{*}}{RT})$$

where *R* is the gas constant and *T* is the absolute temperature. Both $M_{i}^{0}$ and $\Delta G_{i}^{*}$ are composition dependent. In the spirit of the CALPHAD approach, in a multicomponent system, these parameters are expressed with Redlich-Kister-Muggianu polynomials. It is therefore important to state that the composition dependence of the diffusion coefficients arises through both, the thermodynamic factor and mobilities.

In this study, only the isothermal portion of homogenization was considered. Heating was thought to be fast leading quickly to quasi-isothermal stabilized conditions, a reasonable assumption considering the high thermal conductivity of aluminum. Homogenization cooling was studied separately using the KWN model, as presented in the following sections. Due to the complexity of the system of partial differential equations (PDEs) considered, numerical instabilities can arise in the case that numerical integration parameters are not carefully selected. An increased number of phases and components in the system can reduce the stability and possibly the accuracy of the numerical solution. Special attention was given to ensure a reliable integration of the PDEs, by enforcing strict convergence parameters and performing a sensitivity analysis with respect to the simulation time step.

### 3.4. Homogenization Cooling

Although the DGM model can, in principal, predict the reprecipitation of Mg_2_Si during homogenization cooling, it cannot provide information regarding the particle size distribution and associated strengthening. To that end precipitation of Mg_2_Si and strengthening during homogenization cooling were treated with the Kampman-Wagner numerical (KWN) model [42,43]. The major characteristic of the KWN model is that it treats concurrent nucleation, growth and coarsening. Originally the KWN model was developed for isothermal processes. The use of the model in non-isothermal processes is described in [44] while the model was applied for the microstructural evolution and associated strengthening of 6xxx alloys in [45,46].

A binary KWN model was adopted in this work, taking into account the precipitation of the equilibrium phase β-Mg_2_Si and ignoring the precipitation of metastable phases, such as β’. Similar approach has been followed in [47] for the homogenization cooling of a 6005 aluminum alloy and in [48] for the prediction of HAZ hardness of 6061 laser welds. In this work a computational scheme was used in order to implement the model for the continuous cooling following homogenization. The model assumptions were:

1. The diffusion coefficients were considered constant with respect to element concentration.
2. Precipitation was controlled by the diffusion of Mg, as it is the dominant component of Mg_2_Si.
3. The dilution in the matrix was considered infinite.
4. Mg_2_Si particles were considered spherical and with stoichiometric composition.
5. There was no interaction of the diffusion fields around the particles.
6. The cooling rate was slow enough, so that transient diffusion effects were minimal and thus quasi-steady state conditions could be assumed.

Only the basic equations of the model will be presented here. Changes in the Mg_2_Si particle distribution are described by the population balance equation (PBE), which has the following form

(7) $$\frac{\partial n\left(D,t\right)}{\partial t}+\frac{\partial [G\left(D,t\right)n\left(D,t\right)]}{\partial D}=\delta (D-D^{*})I\left(t\right)$$

where n(D,t) represents the number of particles of certain diameter, G(D,t) is the growth/dissolution rate, $D^{*}$ is the critical diameter for nucleation, $\delta (D-D^{*})$ is the Kronecker delta function and I(t) is the nucleation rate. The initial condition is n(D,0)=0 and the boundary conditions are n(0,t)=0 and n(∞,t)=0. The function n(D,t) corresponds to the Mg_2_Si particle size distribution (PSD), which changes continuously during homogenization cooling. Several important characteristic parameters of the PSD, such as the total number of particles, the volume fraction and the mean diameter can be defined. The total number of particles is

(8) $$N=\int_{0}^{\infty }n(D,t)dD$$

The mean particle diameter is

(9) $$\bar{D}=\frac{1}{N}\int_{0}^{\infty }Dn(D,t)dD$$

The volume fraction of the particles is

(10) $$f=\int_{0}^{\infty }D^{3}n(D,t)dD$$

Nucleation of Mg_2_Si can be described by the Becker-Doring theory as discussed in [49]. The theory takes into account an incubation period and the nucleation rate is given by

(11) $$I(t)=N_{0}Z\beta^{*}\exp(-\frac{\Delta G^{*}}{kT})\exp(-\frac{\tau }{t})$$

where $N_{o}$ is the total number of nucleation sites, *Z* is the Zeldovich factor and *k* is the Boltzmann constant. The parameters $\beta^{*}$, $\Delta G^{*}$ and τ are defined as follows: $\beta^{*}$ is the rate at which new atoms are added to the nucleus and is given by

(12) $$\beta^{*}=\frac{4\pi {\left(r^{*}\right)}^{2}\tilde{D}C_{0}}{a^{4}}$$

where $r^{*}$ is the critical radius for nucleation, $\tilde{D}$ is the diffusion coefficient, $C_{0}$ is the nominal composition of the alloy and *a* is the lattice parameter. The energy barrier for nucleation (activation energy) given by

(13) $$\Delta G^{*}=\frac{\Delta G_{0}}{\ln^{2}(\bar{C}/C_{eq})}$$

where $\Delta G_{0}$ is a term encompassing the nucleus/matrix interfacial energy as well as the elastic strain energy of nucleation while the chemical driving force is expressed with the supersaturation ratio $\bar{C}/C_{eq}$ with $\bar{C}$ the mean and $C_{eq}$ the equilibrium concentration of solute (Mg) in the matrix, provided by the equilibrium solvus line of the phase diagram. The incubation time τ is the time needed to achieve steady-state nucleation conditions and is given by

(14) $$\tau =\frac{1}{2\beta^{*}{Ζ}^{2}}$$

Finally the critical radius for nucleation is provided as a function of supersaturation

(15) $$r^{*}=\frac{2\gamma V_{m}}{RT}{\left[\ln(\bar{C}/C_{\mathrm{eq}})\right]}^{-1}$$

where the interface composition is taken equal to the mean composition in the matrix, γ is the nucleus/matrix interfacial energy and $V_{m}$ the molar volume of Mg_2_Si.

The growth/dissolution rate G(D,t) can be calculated by solving the one-dimensional diffusion equation in the radial (*r*) direction for spherical precipitates, assuming a constant diffusion coefficient:

(16) $$\frac{\partial C}{\partial t}=\frac{\tilde{D}}{r^{2}}\frac{\partial }{\partial r}(r^{2}\frac{\partial C}{\partial r})$$

under the assumption that the alloy is dilute enough so that the diffusion fields between particles do not overlap. The initial condition is

(17) $$C(r,0)=\bar{C}$$

The boundary condition at the particle/matrix interface is

(18) $$C(r,t)=C_{i}$$

and at the matrix

(19) $$C(\infty ,t)=\bar{C}$$

where $C_{i}$ is the matrix composition at the precipitate/matrix interface.

The diffusion coefficient is temperature dependent through the relation

(20) $$\tilde{D}={\tilde{D}}_{0}\exp(-\frac{\Delta H^{*}}{RT})$$

where ${\tilde{D}}_{0}$ is the pre-exponential factor and $\Delta H^{*}$ is the activation energy for diffusion.

By considering mass balance, the solution for the growth/dissolution rate for steady-state conditions becomes

(21) $$G=\frac{dr}{dt}=\frac{\bar{C}-C_{i}}{C_{p}-C_{i}}\frac{\tilde{D}}{r}$$

where $C_{p}$ is the solute concentration in the particle (Mg in Mg_2_Si), while the mean solute concentration in the matrix can be calculated by a simple mass balance for the solute concentration

(22) $$\bar{C}=\frac{C_{0}-fC_{p}}{1-f}$$

where $C_{0}$ is the maximum Mg solid solution concentration available for the formation of Mg_2_Si precipitates. 

Finally, coarsening is taken into account by incorporating the dependence of the interfacial composition $C_{i}$ by curvature through the Gibbs–Thomson equation

(23) $$C_{i}=C_{eq}exp\left(\frac{2\gamma V_{m}}{rRT}\right)$$

Regarding the growth/dissolution events, particles with $r>r^{*}$ will grow and particles with $r<r^{*}$ will shrink.

The numerical solution of the PBE involves a discretization of the particle diameter D into a finite number of intervals or classes of equal size *h* in order to obtain the time evolution of the number density of particles at the selected intervals, which for the class ($D_{i}$*,* $D_{i+1}$) is defined as

(24) $$N_{i}=\int_{D_{i}}^{D_{i+1}}n(D,t)dD$$

The PBE then becomes

(25) $$\frac{\partial N_{i}}{\partial t}={-G(D,t)n(D,t)|}_{D_{i+1}}+{G(D,t)n(D,t)|}_{D_{i+1}}+\delta_{ij}I(t)$$

where the value of the product G(D,t)n(D,t) at the interval boundaries for the case of growth with $G(D_{i},t)>0$ is

(26) $${n(D,t)G(D,t)|}_{d_{i}}=\frac{N_{i-1}}{h}G(D_{i},t)$$

and for the case of dissolution with $G(D_{i},t)<0$ is

(27) $${n(D,t)G(D,t)|}_{d_{i}}=\frac{N_{i}}{h}G(D_{i},t)$$

For the strength model, the major strengthening mechanisms contributing to the strength of heat treatable Al-alloys are lattice resistance, strain hardening, Hall-Petch grain boundary strengthening, solution hardening and precipitation hardening. Considering that the first three contributions do not change during homogenization cooling and can be lumped in a single term $\sigma_{0}$, then the yield strength can be written as

(28) $$\sigma =\sigma_{0}+\sigma_{SS}+\sigma_{P}$$

where $\sigma_{ss}$ and $\sigma_{p}$ are, respectively, the contributions from solid solution and precipitation hardening. Solid solution hardening depends on the mean solute concentration in the matrix and can be described by the equation

(29) $$\sigma_{ss}=\sum K_{j}{\bar{C}}_{j}^{2/3}$$

where $K_{j}$ is a constant for each alloying element.

Precipitation strengthening depends on the size and volume fraction of penetrable and non-penetrable particles. Taking as $N_{i}$ the number of particles which belong to a certain class size *i* per unit volume, $r_{i}$ the particle radius of class size *i* and $r_{c}$ the critical radius for the transition between the two mechanisms then for penetrable particles ($r_{i}<r_{c}$) the strength contribution is

(30) $$\sigma_{p}=K_{p}\frac{\sqrt{f}}{\bar{r}}{\left(\frac{\sum_{i}^{n}N_{i}\frac{r_{i}}{r_{c}}}{\sum_{i}^{n}N_{i}}\right)}^{3/2}$$

while for non-penetrable particles ($r_{i}>r_{c}$) the strength contribution is

(31) $$\sigma_{p}=K_{p}\frac{\sqrt{f}}{\bar{r}}$$

The above equations describe the evolution of the yield strength during homogenization cooling from the respective time evolution of the volume fraction and the mean radius of the precipitates Mg_2_Si.

## 4. Results

### 4.1. Microsegregation of As-Cast Alloy

**Figure 2:**
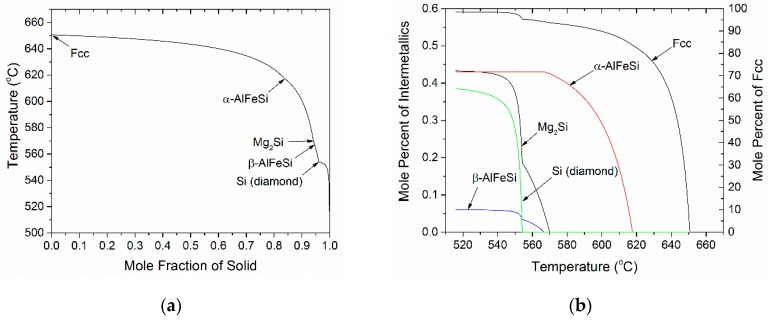

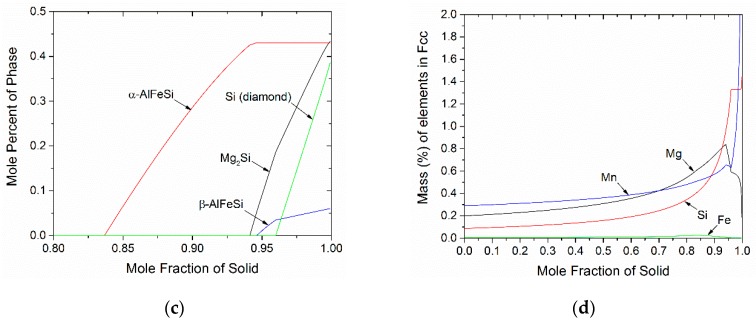
(**a**) Scheil solidification path for 6082 alloy, (**b**) mole percent of phases as a function of temperature, (**c**) mole percent of phases as a function of mass fraction solid and (**d**) mass percent of elements as a function of mass fraction solid during solidification of 6082 alloy.

The Scheil solidification path, for alloy 6082 is shown in Figure 2a as temperature versus mass fraction solid. The phase sequence of formation during solidification is FCC→α-AlFeSi→Mg_2_Si→β-AlFeSi→Si (diamond). The arrows depict the temperature and the mass fraction of solid where each phase starts to form. The mole fraction of phases, formed during solidification, was calculated as a function of temperature and is shown in Figure 2b. Formation of α-AlFeSi starts at 618 °C while further formation of α-AlFeSi stops, when the Mg_2_Si starts to form at 570 °C. The β-AlFeSi phase starts to form at 567 °C and continuous to grow simultaneously with the Mg_2_Si phase until the quaternary eutectic temperature (554 °C) is reached and the remaining liquid transforms to a mixture of FCC, β-AlFeSi, Mg_2_Si and Si (diamond). The phase fractions in the as-cast microstructure are 0.403% for α-AlFeSi, 0.0601% for β-AlFeSi, 0.433% for Mg_2_Si and 0.385% for Si (diamond). The above phases are not distributed uniformly in the microstructure but segregate to regions where the liquid solidifies last, i.e., close to dendrite arm boundaries. This phase segregation is depicted in Figure 2c, which gives the mole percent of all intermetallic phases versus fraction solid. It is seen that the α-AlFeSi phase forms above 80% solidification while all other phases form above 93% solidification. This means that the intermetallic phases form at the secondary dendrite boundaries towards the end of solidification. In this way, Figure 2c can be used to derive the spatial distribution of phases by means of numerical differentiation, with the axis of mass fraction solid corresponding to the distance from the dendrite arm center to the dendrite arm boundary. Accordingly, the microsegregation of the alloying elements in the dendrite arm (FCC matrix) follows a similar trend, i.e., increase towards the end of solidification, as shown in Figure 2d. The drop in the concentration profile of Mg and Fe is attributed to the formation of Mg_2_Si and iron intermetallics respectively.

**Figure 3:**
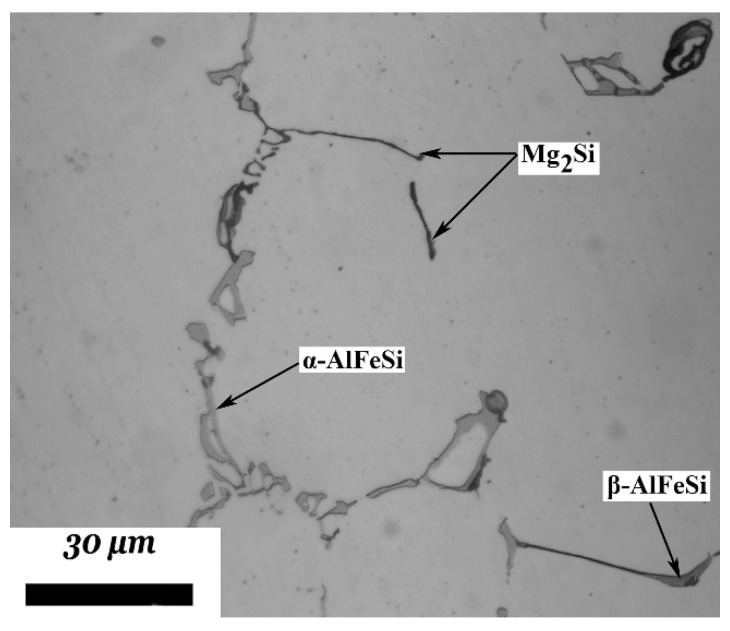
As-cast microstructure of alloy 6082. Reprinted with permission from International Journal of Materials Research, Vol. 105 (2014) pp. 1202–1209 by P.I. Sarafoglou and G.N. Haidemenopoulos, (c) Carl Hanser Verlag GmbH and Co.KG, Muenchen.

The as-cast microstructure of 6082 is depicted in the optical microscopy micrograph of Figure 3. Phase identification has been performed by energy dispersive spectroscopy (EDS) analysis and is discussed in detail in [23]. The microstructure consists of Al-rich solid solution dendrites while the intermetallic phases are segregated at the secondary dendrite arm boundaries. These phases are identified as Mg_2_Si, α-AlFeSi and β-AlFeSi. The intermetallic compound α-AlFeSi appears with a typical lamellar morphology known as “Chinese-script” structure. Following phase identification, the measurement of phase fractions was performed by quantitative metallography using the image analysis software Image J (version 1.50e), after employing low magnification micrographs in order to obtain the most representative microstructure. A comparison between calculated and experimentally-determined phase fractions are shown in Table 3. The calculated phase fractions were derived from Scheil simulations, by careful addition according to the mode of solidification. A similar approach has been followed in [50] to identify optimum Fe/Mn ratios for the elimination of β-AlFeSi from the as-cast microstructure.

**Figure 4:**
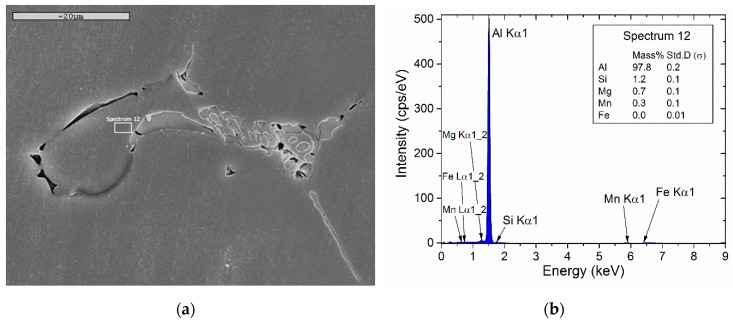
(**a**) SEM micrograph of the as-cast microstructure of alloy 6082, (**b**) EDS analysis of region adjacent to the boundary, indicated by a square in (a).

Since the calculated elemental microsegregation is used as initial condition in the homogenization simulations, it is important to validate the matrix composition, adjacent to the intermetallics, close to the boundary. This is shown in Figure 4, with a SEM microstructure in (a) and the EDS spectrum from the matrix region adjacent to the boundary in (b). The measured compositions are compared against the calculated data from the Scheil simulation of Figure 2d in Table 3. The agreement between measured and calculated compositions is good allowing the elemental microsegregation profiles as well as the phase fraction profiles to be used as initial (as-cast) conditions for the homogenization problem.

### 4.2. Homogenization Holding

**Figure 5:**
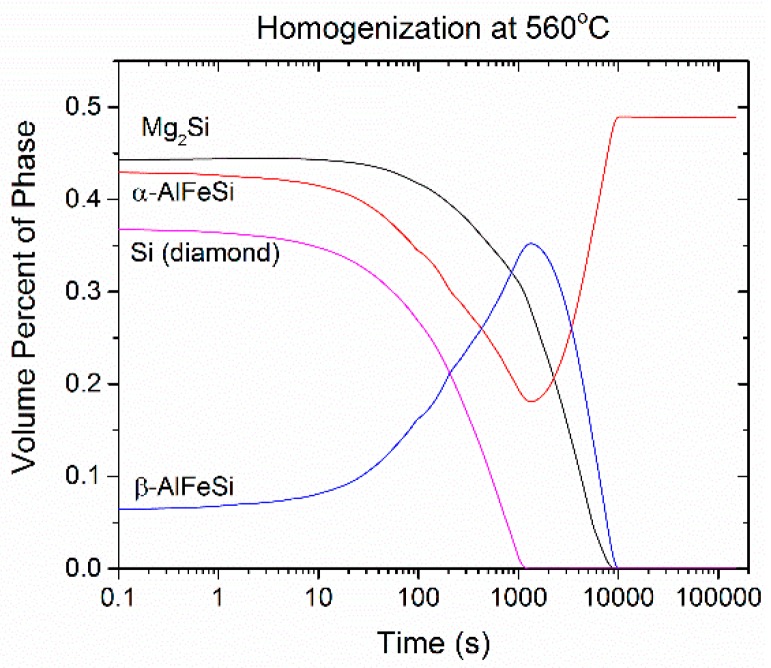
Temporal evolution of volume percent of Mg_2_Si, α-AlFeSi, β-AlFeSi and Si (diamond) during homogenization at 560 °C in 6082 alloy.

**Figure 6:**
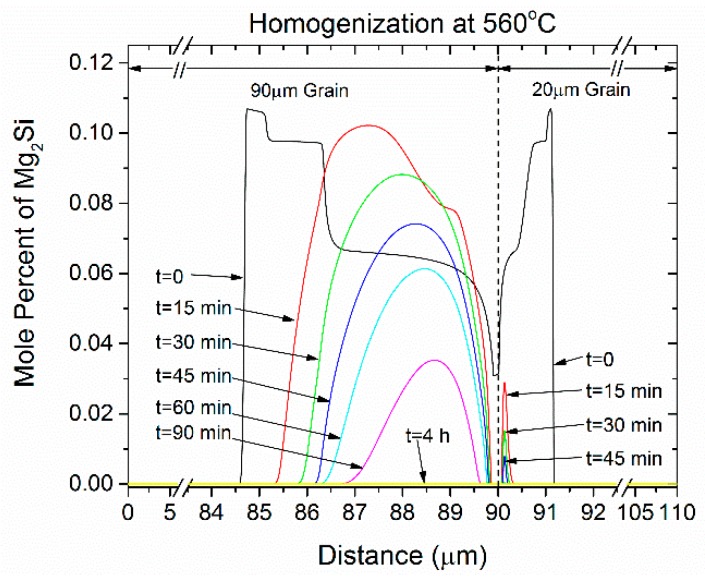
Evolution of spatial distribution of the mole percent of Mg_2_Si at various times during the homogenization of 6082 alloy at 560 °C.

The results of the homogenization Dual Grain Model (DGM) are presented in this section for the 6082 alloy. The evolution of the volume fraction of phases with homogenization time is depicted in Figure 5 for isothermal holding at 560 °C up to 40 h (144,000 s). The Si (diamond) phase dissolves first at the early stages of homogenization, bellow 30 minutes. As it dissolves, excess Si is released, driving the α-AlFeSi→β-AlFeSi transformation and reducing the dissolution rate of Mg_2_Si. Fractions of β-AlFeSi temporarily rise until the Si (diamond) phase disappears and then plummet as α-AlFeSi grows to its equilibrium value, completely consuming the β-AlFeSi. Simultaneously, the dissolution of Mg_2_Si accelerates resulting in its disappearance shortly before β-AlFeSi at approximately 3 h. Similar trends have been reported in the experimental work in [7,10] as well as the modeling works in [25,32]. The reduction of the mole fraction of Mg_2_Si during homogenization at 560 °C is shown in Figure 6. The *t* = 0 curve is the Mg_2_Si profile at the beginning of homogenization (as-cast) as derived from Scheil simulations by numerical differentiation of Figure 2c. As seen in Figure 6, the Mg_2_Si phase dissolves gradually over the course of 3 h at 540 °C. The dissolution of the Mg_2_Si is significantly faster in the small grain (20 μm) due to the shorter diffusion distances. Mg_2_Si fractions rapidly decrease in times bellow 15 min in the small grain, while complete dissolution takes in approximately one hour. In contrast, since the dissolution is controlled by the diffusion of Si and Mg to the grain interior, the kinetics in the larger grain are hindered. Following the complete dissolution of Mg_2_Si during isothermal holding at 560 °C, the Mg_2_Si phase is re-precipitated during cooling from the homogenization temperature in the grain interior. During cooling, the fraction of Mg_2_Si has the potential to reach much higher than the Mg_2_Si present in the as-cast microstructure (at *t* = 0). This is attributed to the additional amount of Si arising from the dissolution of the Si (diamond) phase and the β-AlFeSi→α-AlFeSi transformation during homogenization, since the α-AlFeSi phase is leaner in Si than the β-AlFeSi phase. Thus, there is more Mg and Si available in the matrix to form Mg_2_Si during homogenization cooling. The precipitation of Mg2Si during homogenization cooling will be discussed further in the next section.

**Figure 7:**
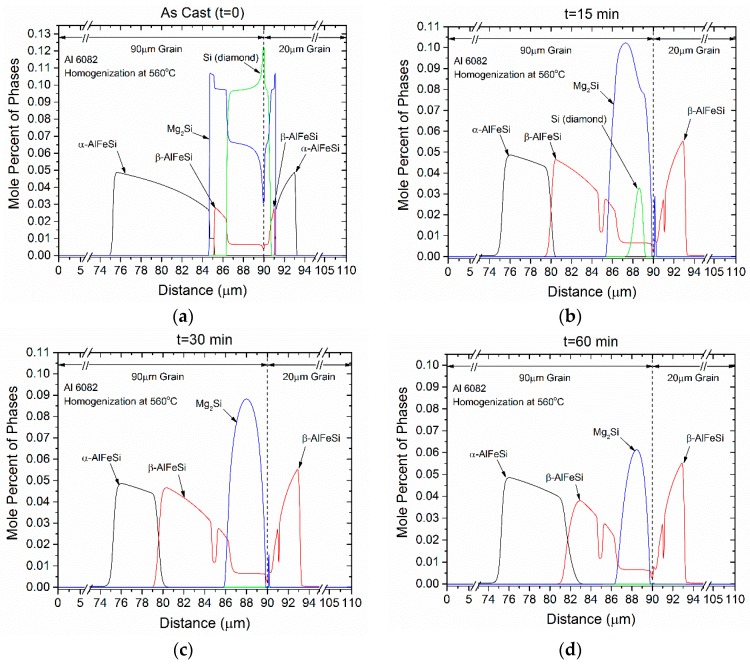

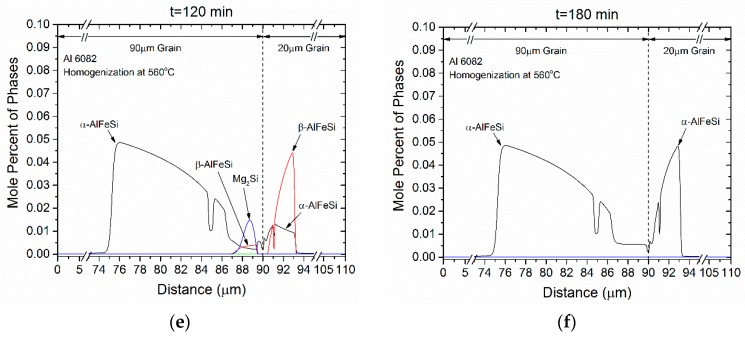
Spatial evolution of α-AlFeSi, β-AlFeSi, Mg_2_Si and Si (diamond) during homogenization at 560 °C of 6082 alloy: (**a**) As Cast, (**b**) 15 min, (**c**) 30 min, (**d**) 60 min, (**e**) 2 h (120 min), and (**f**) 3 h (180 min).

The spatial evolution of the transformations taking place during homogenization are depicted in Figure 7a–f for times 0 (as-cast), 15, 30, and 60 min, as well as 2 h (120 min) and 3 h (180 min). The following observations can be made:

- Si (diamond) rapidly dissolves, as it is consumed by Mg_2_Si that grows in the same location near the grain boundary. Simultaneously Mg_2_Si gradually dissolves from the grain interior to the boundary as Si and Mg migrate to the grain center. The excess Si released from the dissolution of the Si (diamond) phase, causes β-AlFeSi to grow against α-AlFeSi at the early stages of homogenization. After the complete dissolution of Si (diamond), the transformation reverses, as α-AlFeSi gradually consumes β-AlFeSi completely.
- The profiles of α-AlFeSi and β-AlFeSi exhibit an exact spatial correspondence. Where there is a drop in the fraction of β-AlFeSi, there is a rise in the fraction of α-AlFeSi and vice versa, at the same location.
- The phase fraction profiles are steeper, and the transformation is faster in the smaller grain. Yet the kinetics of β-AlFeSi→α-AlFeSi transformation in the small grain were severely hindered since α-AlFeSi was completely consumed during the early stages. A nucleation event is required to regrow α-AlFeSi, effectively reducing the rate of the transformation.

The above results indicate that the β-AlFeSi→α-AlFeSi transformation does not take place immediately during homogenization. The inverse transformation α-AlFeSi→β-AlFeSi takes place first, driven by the dissolution of Si (diamond) and Mg_2_Si. It is followed by the forward transformation β-AlFeSi→α-AlFeSi at a critical time that coincides with the disappearance of Si (diamond). All transformations considered occur at a region adjacent to the grain boundary. In addition, all transformations except α-AlFeSi→β-AlFeSi take place at considerably increased rates in the smaller grains. The exception originates from the complete dissolution of α-AlFeSi in small grains, requiring nucleation and growth to drive the transformation to completion.

**Figure 8:**
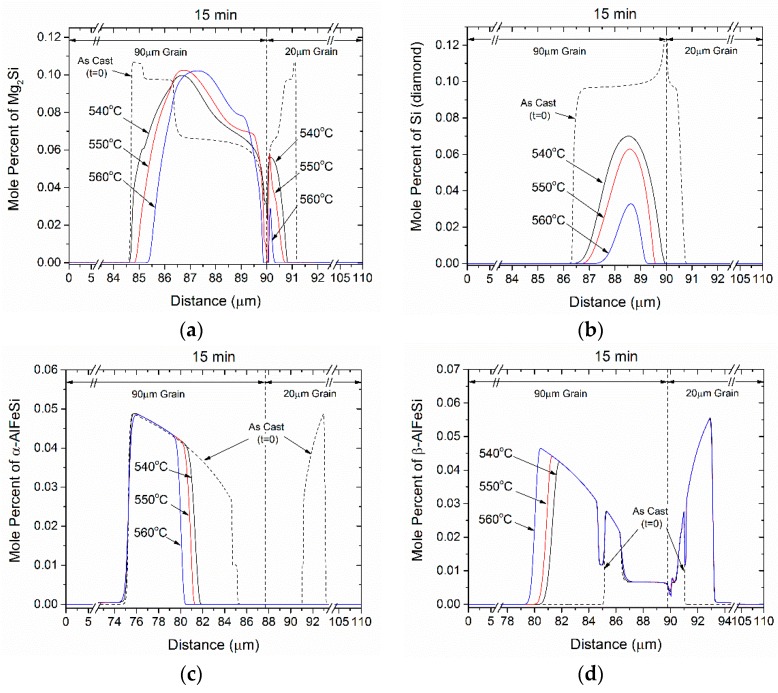
Spatial distribution of the mole percent of (**a**) Mg_2_Si, (**b**) Si (diamond), (**c**) α-AlFeSi, and (**d**) β-AlFeSi after homogenization of 6082 alloy for 15 min at various temperatures (540, 550 and 560 °C).

While grain size influences the transformation kinetics through the distance over which diffusion of alloying elements takes place, homogenization temperature influences the diffusion rate. For the 6082 alloy the spatial distribution of intermetallic phases is depicted in Figure 8a–d for 3 different homogenization temperatures, 540, 550 and 560 °C for homogenization time *t* = 15 min. As expected, the dissolution rate of Mg_2_Si and Si (diamond) significantly increase at elevated temperatures. Additionally, the α-AlFeSi→β-AlFeSi transformation, taking place at early homogenization stages also accelerates with an increase in temperature. Subsequently, transient transformation effects decay quicker and the forward α-AlFeSi→β-AlFeSi transformation can start earlier and progress faster. Increased homogenization temperature increase both the driving forces for Mg_2_Si, Si (diamond) and β-AlFeSi dissolution, as well as the diffusivities of the components in the matrix, effectively accelerating the diffusional transformations.

**Figure 9:**
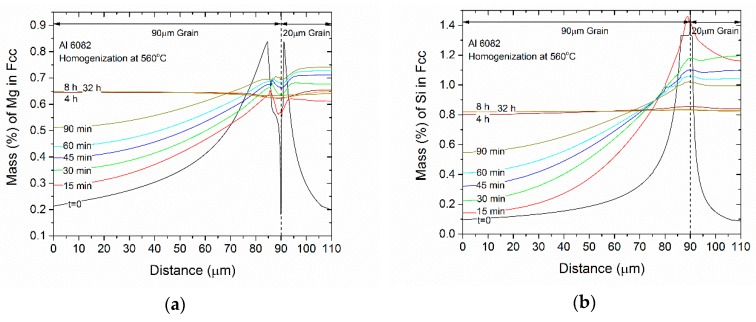

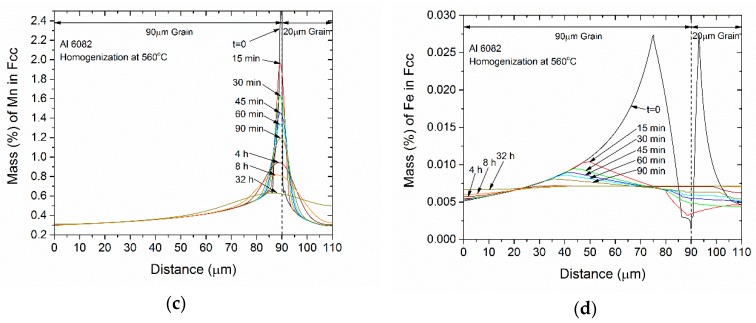
Evolution of concentration profiles during homogenization at 560 °C of 6082 alloy, (**a**) Mg, (**b**) Si, (**c**) Mn, and (**d**) Fe.

As stated in the introduction, one of the aims of homogenization is the removal of microsegregation, which is present in the as-cast microstructure. The evolution of the concentration profiles of Mg, Si, Mn, and Fe in the FCC phase during homogenization at 560 °C, as predicted by the DGM model, is given in Figure 9. The homogenization times considered are 15, 30, 60, and 90 min, as well as 4, 8, and 32 h. The *t* = 0 curve corresponds to the as-cast microsegregation profile (Scheil profile). The following remarks can be made:

- The concentration profiles for Mg, Si and Mn become more uniform with homogenization time. The profile for Fe in FCC change mostly due to its absorption from the formation of α-AlFeSi and β-AlFeSi since the diffusivity of Fe in Al is very low.
- The average concentration of Mg and Si in the matrix interior (away from the boundary) increases with homogenization time, especially in the smaller grain.
- The profiles homogenize faster in the smaller grain due to a shorter diffusion distance.
- The compositional fluctuations close to the boundary are due to the dissolution of Mg_2_Si, Si (diamond) and the β-AlFeSi→α-AlFeSi transformation. These fluctuations decay with homogenization time.
- The spatial evolution of the concentration profiles is consistent with the spatial evolution of the phase fractions.

**Figure 10:**
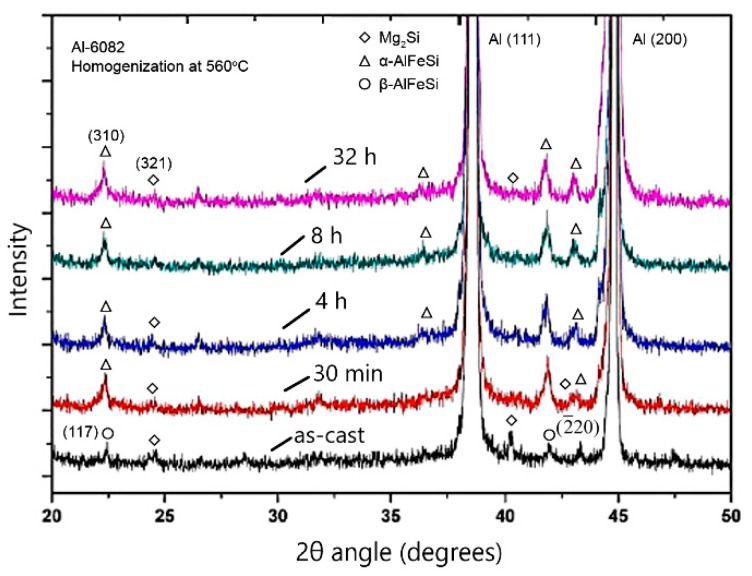
X-ray diffraction spectra after homogenization at 560 °C for 0.5, 4, 8, and 32 h, indicating dissolution of Mg_2_Si and transformation of β-AlFeSi to α-AlFeSi. Alphabetical Indexes for experimental patterns, International Center for Diffraction Data (ICCD). α-AlFeSi: PDF #01-071-4015; β-AlFeSi; PDF #41-0894; Mg2Si: PDF#35-0773.

The β-AlFeSi→α-AlFeSi transformation was monitored experimentally with XRD. For the 6082 alloy an XRD spectrum was taken after 0.5, 4, 8, and 32 h homogenization at 560 °C and cooling to room temperature. The as-cast condition was also analyzed with XRD. The results appear in Figure 10. In the caption of the figure the number of the corresponding powder diffraction files (PDF) of the International Center for Diffraction Data (ICCD) are given and the reference is quoted. Diffraction peaks corresponding to the aluminum matrix, Mg_2_Si, α-AlFeSi and β-AlFeSi are evident. The β-AlFeSi peaks are replaced by α-AlFeSi peaks in the 0.5 h spectrum. This does not mean that the transformation is completed in 0.5 h. It is more probable that at 0.5 h the fraction of the β-AlFeSi phase is small and beyond the detection limit of the XRD. The results are in general agreement with the XRD results reported in [21] for an Al-0.602Mg-0.98Si-0.427Mn-0.222Fe alloy homogenized at 560 and 580 °C.

**Figure 11:**
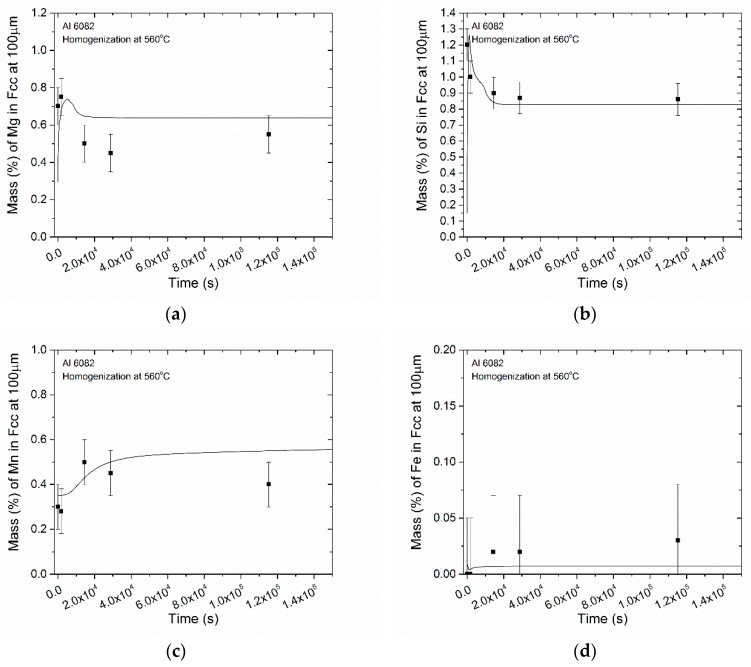
Comparison of DGM model prediction with EDS experimental data regarding the evolution of matrix concentration adjacent to the boundary during homogenization of 6082 alloy, (**a**) Mg, (**b**) Si, (**c**) Mn, (**d**) Fe.

In order to experimentally validate the DGM predictions regarding the removal of microsegregation, EDS analysis at several homogenization times was performed, in the matrix close to the boundary. The results for Mg appear in Figure 11a indicating an initial enrichment of the matrix near the boundary in Mg, followed by a gradual depletion until equilibrium is reached. Simulation results agree with experimental evidence, though some discrepancies are apparent. More specifically, the DGM overestimates Mg concentration at intermediate homogenization times (4 and 8 h). The deviation was mostly attributed to inconsistences in the distance of the measured points from the grain boundary, contributing to experimental error. The results for Si appear in Figure 11b. The evolution of Si concentration with homogenization time predicted by the DGM agrees with experimental evidence, as the solid line lays well inside the measurement error bars. Si concentration adjacent to the grain boundary steadily decrease as microsegregation is eliminated through diffusion. The results for Mn appear in Figure 11c. The DGM model predicts well the Mn concentration in the matrix at short homogenization times, but over estimates its values at longer times. This can be attributed to uncertainties associated with accelerated diffusion phenomena as grain boundary diffusion, as well as due to Mn retention in the α-AlFeSi phase. The results for Fe appear in Figure 11d. Due to the low diffusivity of Fe in Al, the Fe concentration does not change significantly with homogenization time. The simulation results are in good agreement with the experimental data.

### 4.3. Homogenization Cooling

**Figure 12:**
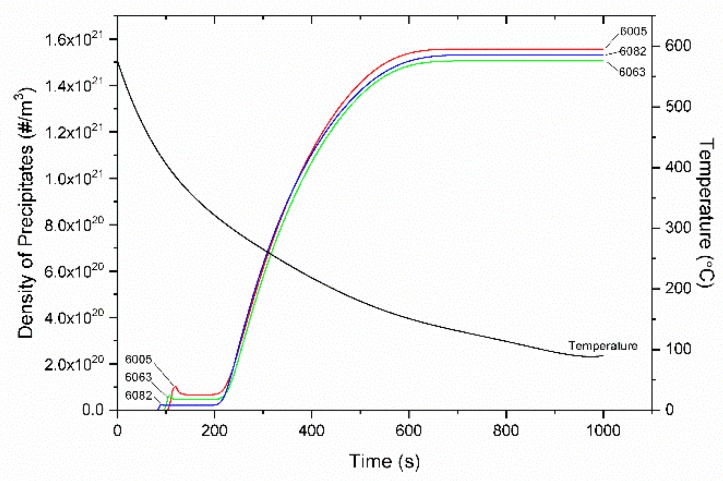
Variation of the number density of Mg_2_Si precipitates with time during homogenization cooling for 6063, 6005 and 6082 alloys. The cooling curve is also indicated.

**Figure 13:**
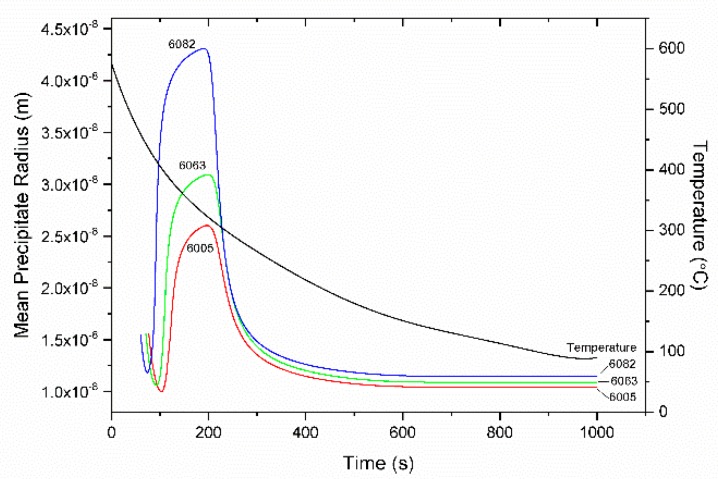
Variation of the mean particle radius of Mg_2_Si precipitates with time during homogenization cooling for 6063, 6005 and 6082 alloys. The cooling curve is also indicated.

The KWN model for the precipitation of Mg_2_Si, described in Section 3.4, was applied in 6063, 6005, and 6082 alloys. The cooling curve considered, after homogenization at 580 °C, was determined with a thermocouple attached at the specimen during cooling. The cooling curve is plotted together with the simulation results in the following figures. The values of parameters used in the KWN and strength models are shown in Table 4. The variation of the particle number density is plotted together with the cooling curve in Figure 12. After a first nucleation event at 80–100 s, the particle number density increases rapidly following a second nucleation event, which starts at 200 s and ends at 600 s, where the number density stabilizes. The variation of mean particle radius $\bar{r}$, is depicted in Figure 13. After the first nucleation event, the mean particle radius increases due to growth, reaching a maximum at 200 s at the onset of the second nucleation event. The new smaller particles nucleated cause the decrease of the mean particle radius. This is because the new particles have a continuously decreasing critical radius $r^{*}$ due to the corresponding increase of supersaturation. However, growth slows down with the drop in temperature. At t = 600 s, where the second nucleation event is completed and no further particles form, the mean radius $\bar{r}$ stabilizes as further growth is not feasible below this temperature due to limited diffusion. 

**Figure 14:**
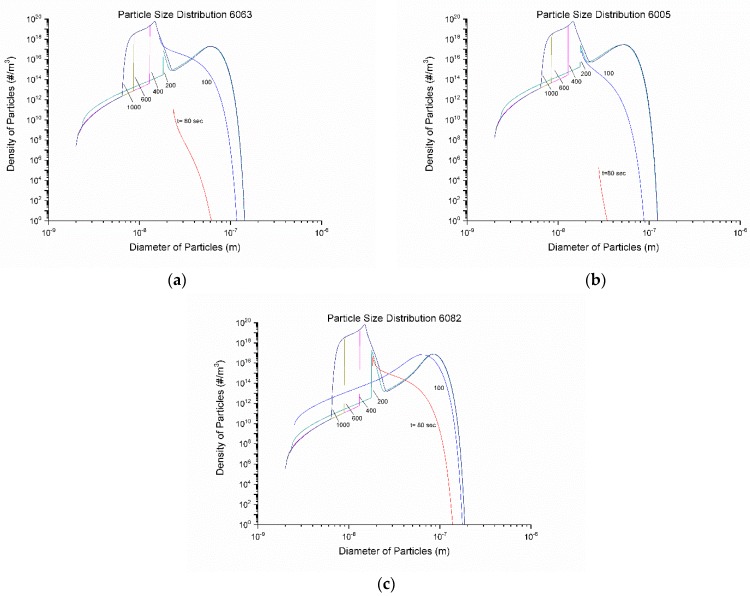
Evolution of particle size distributions (PSD) of Mg_2_Si precipitates with time during homogenization cooling, (**a**) 6063, (**b**) 6005, and (**c**) 6082 alloy.

**Figure 15:**
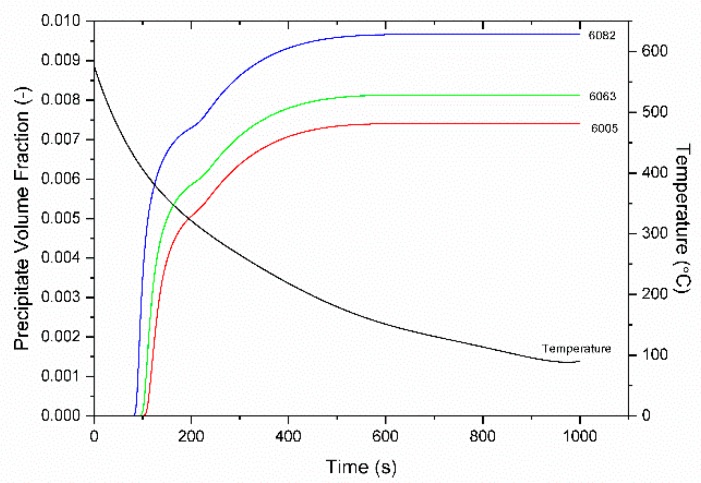
Evolution of the volume fraction of Mg_2_Si precipitates with time during homogenization cooling for 6063, 6005, and 6082 alloys. The cooling curve is also indicated.

The particle size distributions (PSD) are shown in Figure 14a–c for 6063, 6005, and 6082 alloys respectively. During the first nucleation event, at 80–100 s particles of smaller diameters continue to precipitate while the previously precipitated particles grow. This results in a broadening of the PSD curve to the right. At 200 s the onset of the second nucleation event corresponds to the peak that begins to form at approximately 2 × 10^−8^ m diameter of the PSD. This peak continues to grow and move to the left as the second nucleation event continues, and smaller particles precipitate. This can also be seen in the $\bar{r}$ graph of Figure 13, where the mean particle radius continues to decrease. Growth no longer takes place because of the decreased temperature and that is why the PSD curves no longer move to the right. The bi-modal nature of the PSD arises from the two nucleation events discussed above. Similar multi-modal PSD curves have been reported recently in [32] and have been attributed to multiple nucleation events, which arise from differences in local chemistry. The evolution of the volume fraction of the particles is depicted in Figure 15. The knee in the curves at 200 s corresponds to the onset of the second nucleation event. The volume fraction of the particles stabilizes at 600 s, which corresponds to the stabilization of the mean radius $\bar{r}$. The highest volume fraction is obtained for alloy 6082.

**Figure 16:**
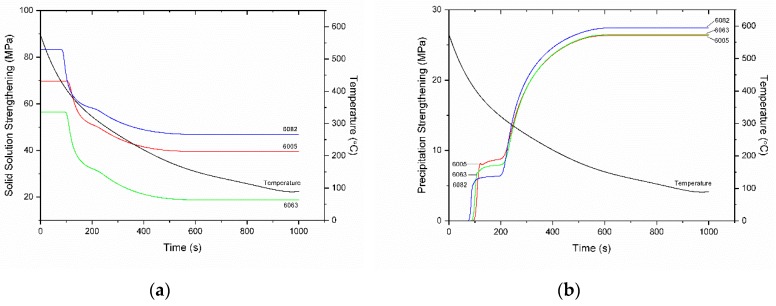
Evolution of solid solution strengthening in (**a**) and precipitation strengthening in (**b**) during homogenization cooling for 6063, 6005, and 6082 alloys. The cooling curve is also indicated.

**Figure 17:**
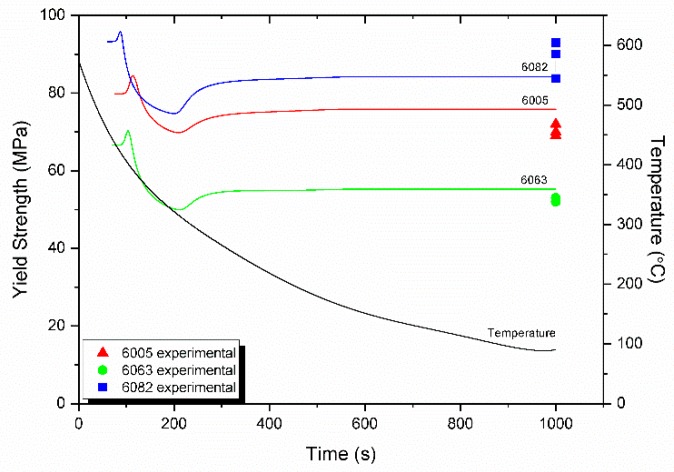
Evolution of the yield strength during homogenization cooling and comparison with experimental data for 6063, 6005, and 6082 alloys.

Regarding strengthening during homogenization cooling, as stated in Section 3.4 only the solid solution strengthening (SSS) and precipitation strengthening (PS) contributions vary during cooling. These variations are depicted in Figure 16. Before the first nucleation event there is only SSS contribution since all Mg and Si are dissolved in the matrix as a result of the Mg_2_Si dissolution during homogenization holding. Alloy 6082 has the largest SSS contribution due to the higher amount of both Mg and Si than the other two alloys. The SSS contribution falls and at the same time the PS contribution rises during the first nucleation event at 80–100 s. The slight differences of the onset of PS between the three alloys are due to differences in the development of supersaturation and the respective driving force for nucleation. The knee at the SSS curves corresponds to the onset of the second nucleation event. The SSS contribution continues to decrease while the PS contribution increases. Both contributions stabilize roughly at 600 s. The evolution of the overall yield strength of the alloys during homogenization cooling is depicted in Figure 17 where it is compared against experimental data. Tensile properties are given in Table 5. The model slightly overestimates the yield strength of 6013 and 6005 by 10%. Taking into account that the model is based on a binary KWN approximation, the agreement between model and experiment is considered satisfactory.

## 5. Conclusions

The presented modeling approach treats three consecutive steps of the homogenization process chain. Microsegregation during solidification, dissolution of Mg_2_Si, Si (diamond) and β-AlFeSi (β-Al_5_FeSi) to α-AlFesi (α-Al_12_(FeMn)) transformation during homogenization holding as well as Mg_2_Si precipitation and associated strengthening during homogenization cooling have been modeled with a CALPHAD-based multicomponent diffusion model and the Kampmann–Wagner Numerical (KWN) precipitation framework. The major conclusions are as follows:

- A Dual-Grain Model (DGM) has been developed to describe the effect of grain size inhomogeneity during homogenization of 6082 Al alloy. The transformation of β-AlFeSi to α-AlFeSi proceeds in two steps. First, the reverse transformation takes place as β-AlFeSi grows against α-AlFeSi due to the excess Si released from the dissolution of the Si (diamond) phase. The forward transformation of β to α-AlFeSi commences once dissolution of the Si (diamond) phase is completed.
- During the transformation, the fractions of α-AlFeSi and β-AlFeSi exhibit an exact spatial and temporal correspondence. The DGM model predictions are in good agreement with experimental data.
- The KWN precipitation model was applied to the binary Al-Mg_2_Si system in 6005, 6063, and 6082 Al alloys. The model indicated the development of a bimodal particle size distribution during homogenization cooling, arising from corresponding nucleation events. The associated strengthening arises from contributions due to solid solution and precipitation strengthening and is in good agreement with experimental results.
- The proposed modeling approach is a valuable tool for the prediction of microstructure evolution during the homogenization of extrudable 6xxx aluminum alloys, including the often-neglected part of homogenization cooling.

## Figures and Tables

**Table 1:** Alloy compositions (wt%). Al is balance composition.

Al-Alloy	Mg	Si	Fe	Mn
6063	0.5346	0.4194	0.1895	0.0311
6005	0.4896	0.686	0.1552	0.2227
6082	0.63	0.9	0.2	0.45

**Table 2:** Models used in the present work.

Process	Phenomena Modeled	Relevant Models
Casting/Solidification	Microsegregation of elements and phases	Scheil-Gulliver
Homogenization (holding)	Dissolution of Mg_2_Si Transformation of β-AlFeSi to α-Al(FeMn)Si	Multicomponent, multiphase diffusion—Dual Grain Model (DGM)
Homogenization (cooling)	Precipitation of Mg_2_Si Precipitation Strengthening	Kampmann-Wagner Numerical (KWN) precipitation model—Strength model

**Table 3:** Fractions of phases and matrix composition close to the boundary in as-cast 6082.

**Phase Fractions**	**Calculated (%)**	**Measured (%)**
β-AlFeSi + α-AlFeSi	0.587	0.607
Mg_2_Si	0.313	0.365
Quaternary Eutectic	0.411	0.41
**Matrix Composition Close to Boundary**	**Calculated (mass%)** **(at 0.92 Fraction of Solid)**	**Measured (mass%)**
Si	1.33	1.2 (σ = 0.1)
Mg	0.6	0.7 (σ = 0.1)
Mn	0.63	0.3 (σ = 0.1)
Fe	0.003	0 (σ = 0.01)
Al	97.4	97.8 (σ = 0.2)

**Table 4:** Parameters used in the KWN and strength models for homogenization cooling.

Parameter	Symbol	Value	Source
Concentration of Mg in Mg_2_Si	$C_{p}$ (wt%)	63.4	Stoichiometry
Pre-exponential term for diffusion of Mg	$D_{o}$ (m^2^/s)	2.2 × 10^−4^	[44]
Activation energy for diffusion of Mg	$\Delta H^{*}$(J/mol)	130,000	[44]
Nucleus/matrix interfacial energy (Mg_2_Si)	γ (J/m^2^)	0.5	[51]
Molar volume Mg_2_Si	$V_{m}$ (m^3^/mol)	3.95 × 10^−5^	[51]
Total bulk nucleation site density	$N_{o}$ (#/m^3^)	10^19^–10^20^	Adjustable parameter
Dimensionless term in activation energy for nucleation	$\Delta G_{o}$	4 × 10^−19^	[42]
Transition radius for precipitation strengthening	$r_{c}$ (m)	5 × 10^−9^	[52]
Constant contribution to yield strength	$\sigma_{o}$ (MPa)	10	[52]
Parameter for solid solution strengthening of Si	$K_{Si}$	66.3	[53]
Parameter for solid solution strengthening of Mg	$K_{Mg}$	29	[53]
Parameter for precipitation hardening	$K_{p}$	4 × 10^−6^	[42]

**Table 5:** Tensile testing results for the 6005, 6063, and 6082 Al alloys.

Alloy	Specimen	σ_yield_ (ΜPa)	σ_yield_ Mean (MPa)	ε_fracture_ (%)
6005	1st	72	70.33	27.7
	2nd	69		25
	3rd	70		26.3
6063	1st	52	52.33	33.3
	2nd	53		30.5
	3rd	52		30.5
6082	1st	83.75	88.91	16.6
	2nd	90		15.2
	3rd	93		16.6
